# Formation of a Stable Co-Amorphous System for a Brick Dust Molecule by Utilizing Sodium Taurocholate with High Glass Transition Temperature

**DOI:** 10.3390/pharmaceutics15010084

**Published:** 2022-12-27

**Authors:** Shohei Aikawa, Hironori Tanaka, Hiroshi Ueda, Masato Maruyama, Kazutaka Higaki

**Affiliations:** 1 Department of Pharmaceutics, Faculty of Pharmaceutical Sciences, Okayama University, Okayama 700-8530, Japan; 2 Formulation Research Department, Formulation R&D Laboratory, Shionogi & Co., Ltd., Hyogo 660-0813, Japan; 3 Bioanalytical, Analysis and Evaluation Laboratory, Shionogi & Co., Ltd., Osaka 561-0825, Japan

**Keywords:** amorphous, co-amorphous, crystallization, sodium taurocholate, glass transition temperature, intermolecular interaction, dissolution testing

## Abstract

Brick dust molecules are usually poorly soluble in water and lipoidal components, making it difficult to formulate them in dosage forms that provide efficient pharmacological effects. A co-amorphous system is an effective strategy to resolve these issues. However, their glass transition temperatures (*Tg*) are relatively lower than those of polymeric amorphous solid dispersions, suggesting the instability of the co-amorphous system. This study aimed to formulate a stable co-amorphous system for brick dust molecules by utilizing sodium taurocholate (NaTC) with a higher *Tg*. A novel neuropeptide Y_5_ receptor antagonist (AntiY_5_R) and NaTC with *Tg* of 155 °C were used as the brick dust model and coformer, respectively. Ball milling formed a co-amorphous system for AntiY_5_R and NaTC (AntiY_5_R-NaTC) at various molar ratios. Deviation from the theoretical *Tg* value and peak shifts in Fourier-transform infrared spectroscopy indicated intermolecular interactions between AntiY_5_R and NaTC. AntiY_5_R-NaTC at equal molar ratios resulting in an 8.5-fold increase in AntiY_5_R solubility over its crystalline form. The co-amorphous system remained amorphous for 1 month at 25 °C and 40 °C. These results suggest that the co-amorphous system formed by utilizing NaTC as a coformer could stably maintain the amorphous state and enhance the solubility of brick dust molecules.

## 1. Introduction

Small molecules remain important as a therapeutic modality owing to more than half of their approval rate on the total new molecular entities (NMEs) by the US Food and Drug Administration (FDA) [1]. However, improvement in their dissolution property is often required because approximately 75% of new drug candidates are poorly water-soluble [2]. These drugs cannot provide dissolved concentrations high enough in gastrointestinal fluids, resulting in low bioavailability [2,3]. Particularly, “brick dust molecules” with low solubility in both aqueous and lipoidal vehicles and melting points > 200 °C are drugs with very low aqueous solubility (e.g., less than 1 wt %) [4,5,6] and are difficult to prepare as lipid-based formulations (LBF) [7]. Because a high melting point should be derived from high crystallinity and high intermolecular forces in the solid state [4], disruption of these intermolecular forces is considered a method to overcome the poor solubility and enhance the water solubility of drug candidates.

To enhance drug solubility, conversion of the crystal form and formulation design have been widely investigated [8,9,10,11]. Amorphization from crystal forms, with disruption of crystallinity and intermolecular forces, is a promising approach to significantly enhance drug solubility and oral absorption due to the higher energy state via randomly arranged molecules [12,13]. However, a higher energy state causes thermodynamic instability with molecular mobility, resulting in low physical stability. The tendency towards reorganization into an ordered crystal lattice during manufacturing or storage poses a challenge in practical use.

Polymeric amorphous solid dispersion (PASD) addresses such issues as a common strategy to reduce molecular mobility and maintain the amorphous state [8,12,13,14,15,16]. In PASD, the amorphous state of drugs is formed through intermolecular interactions with a polymer, resulting in a higher glass transition temperature (*Tg*). The increase in *Tg* of PASD is usually associated with a reduction in its molecular mobility and stabilization against recrystallization. Polyvinylpyrrolidone, polyvinyl alcohol, aminoalkyl methacrylate copolymers, cellulosic polymers, and their derivatives are often used for preparing PASD [14,15,16,17,18,19,20,21]. Evaporation, ball milling, spray drying, and hot-melt extrusion (HME) have been employed as preparation methods for PASD [22,23]. HME is preferable due to its solvent-free technology [24], as environmental pollution is a growing concern. A drawback to the use of HME is the difficulty in its application for brick dust molecules because the degradation temperatures of polymers used as solid dispersion carriers are often around 200 °C [25,26], which is lower than the melting point of brick dust molecules. Ball milling, which does not require elevated temperatures, has been reported as a PASD preparation method for drugs with high melting points [27]. PASD formulations generally require a large amount of polymer to enhance drug solubility, resulting in low drug loadings limited to approximately 20–30 wt %. This is a disadvantage for both usability and manufacturing [28,29]. In addition, PASD formulations potentially cause phase separation and/or recrystallization owing to the hygroscopic nature of the polymers [30].

On the other hand, in recent years, co-amorphous systems for drugs designed using a combination with small-molecule pharmaceutical excipients instead of polymers have gained greater attention as alternatives to PASD for amorphization technique [31,32,33]. The co-amorphous system is considered to stabilize the amorphous state of a drug with one or more low-molecular-weight pharmaceutical excipients such as amino acids, organic acids, and other small-molecule drugs [28], and they usually form a homogeneous amorphous phase [28,34,35]. It has been reported that the co-amorphous system can generally increase drug loading to 50 wt %, which is 2–3 times larger than polymer-based PASD [28]. However, *Tg* values of co-amorphous systems tend to be lower than those of PASD because the co-amorphous systems are usually not composed of polymers, whose *Tg* values are approximately 50–150 °C [25], but low-molecular-weight excipients, whose *Tgs* are approximately −60–70 °C [29,36,37]. Therefore, increasing the *Tg* of co-amorphous systems is important for preparing co-amorphous systems that stably maintain the amorphous state of the drugs. To develop a stable co-amorphous system and apply it to pharmaceutical formulations for various drugs, it is necessary to explore adequate coformers with a high *Tg*; however, there are limited studies on increasing the *Tg* of co-amorphous systems with coformers [35,38,39].

Sodium taurocholate (NaTC) is a promising candidate for the formation of a stable co-amorphous system with drugs because its chemical formula is similar to that of cholic acid, a bile acid with a high *Tg* (120 °C), and one of the coformers generating amorphous forms [39]. Previously, it was reported that NaTC generated co-amorphous forms for several active pharmaceutical ingredients (API), including brick dust molecules, at a molar ratio of 1:1, resulting in significant enhancement of dissolution and solid-state stability. However, the details of the generated co-amorphous forms, such as the strength of the intermolecular interactions between API and NaTC based on the *Tg* values, have never been investigated due to an inability to determine the *Tg* of pure NaTC [40,41]. In this study, we aimed to utilize NaTC as a possible coformer to generate a co-amorphous system of a brick dust compound by ball milling. We investigated the details of the co-amorphous system prepared by measuring the *Tg* of pure NaTC, and evaluated the improvement in dissolution rate and recrystallization tendency. A novel neuropeptide Y_5_ antagonist (AntiY_5_R), originally developed by Shionogi & Co., Ltd., was selected as the model brick dust molecule because of its low solubility and high melting point (250 °C). Co-amorphous systems of AntiY_5_R with NaTC at various compositions were subjected to thermal and spectroscopic analyses, in vitro dissolution tests, and physical stability tests under long-term and accelerated conditions [42].

## 2. Materials and Methods

### 2.1. Materials

A novel neuropeptide Y_5_ receptor antagonist (AntiY_5_R), N-(((1r,4r)-4-((6-fluorobenzo[d]oxazol-2-yl)amino)cyclohexyl)methyl)-2-methylpropane-2-sulfonamide, was designed by Shionogi & Co. Ltd. (Osaka, Japan). Sodium taurocholate (NaTC) was purchased from Fujifilm Wako Pure Chemical Corporation (Osaka, Japan). All other reagents used were analytical-grade commercial products.

### 2.2. Thermogravimetry/Differential Thermal Analysis

Thermogravimetry/differential thermal analysis (TG/DTA) of AntiY_5_R and NaTC was performed using a STA7200RV instrument (Hitachi High-Tech Science Corporation, Tokyo, Japan) to determine the thermal degradation point. A total of 2–6 mg of the sample was placed in an aluminium pan and heated at 10 °C/min to 350 °C. Changes in sample weight were recorded as a function of temperature. The data were analyzed using the TA7000 standard analysis version 11.2 (Hitachi High-Tech Science Corporation).

### 2.3. Solubility

To determine the solubility of AntiY_5_R in distilled water, 0.1 N HCl (pH 1.2), and phosphate buffer (pH 6.8), approximately 10 mg of AntiY_5_R was added to 20 mL of each solvent and shaken at 70 cycles/min in a MM-10 water bath shaker (TAITEC, Koshigaya, Saitama, Japan) at 37 °C for 24 h. A portion of the test media was filtered through a 0.45 mm syringe filter (GL Sciences Inc., Tokyo, Japan). A 1 mL sample of the filtered solution was analyzed using a high-performance liquid chromatography (HPLC) system, which consisted of a Quaternary Solvent manager as the pump system, Sample Manager-FTN as an autosampler, and a TUV detector set at 300 nm (Waters ACQUITY UPLC H-Class system, Tokyo, Japan). The analytical column was an ACQUITY UPLC BEH C18, 1.7 μm 2.1 × 50 mm, and the mobile phase, 0.1% trifluoroacetic acid: acetonitrile = 60:40, was delivered at 0.4 mL/min at 35 °C. The standard curves of 1–100 μg/mL showed a coefficient of variation ranging from 0.13 to 7.6% and correlation coefficients greater than 0.9999.

### 2.4. Preparation of Physical Mixtures of AntiY_5_R and Sodium Taurocholate

Physical mixtures of AntiY_5_R and NaTC, with a total weight of 100 mg, were prepared by mixing the components in several molar ratios in a mortar and pestle for approximately 1 min. A correction for the weight of absorbed water in NaTC was made from the TG results. The mixture of AntiY_5_R with NaTC in the molar ratio *X*:*Y* is represented as AntiY_5_R-NaTC (*X*:*Y*) in this study.

### 2.5. Preparation of Co-Amorphous Systems of AntiY_5_R and Sodium Taurocholate

Co-amorphous systems of AntiY_5_R and NaTC were prepared by ball milling. AntiY_5_R and NaTC were weighed at various molar ratios (100 mg total). The loading weight of NaTC was adjusted by correcting the weight of the absorbed water, which was analyzed by TG. The mixture was weighed in a 2 mL aluminium vial containing two tungsten balls (6 mm diameter) using a SHAKE MASTER NEO (Bio Medical Science corporation, Tokyo, Japan) in a cold room at 4 °C, and ball milling was performed at 100 rpm for 180 min to obtain a fully co-amorphous system [43].

### 2.6. X-ray Powder Diffraction

X-ray powder diffraction (XRPD) analysis was performed using a SmartLab diffractometer (Rigaku Corporation, Tokyo, Japan) equipped with a 9 kW rotating anode using Cu Kα radiation (λ = 1.54186 Å) and a HyPix-3000 detector. The sample was placed in a hole (diameter, 3 mm; depth, 0.1 mm) in an aluminium plate and smoothed using a spatula. The distance between the sample and the detector was 331 mm, and the diffractometer was equipped with a cross-beam optic (CBO), providing a parallel beam. Using a parallel-slit collimator with 2.5° collimation and a slit of 0.05 mm height and 0.5 mm width, the beam footprint for all measurement configurations was smaller than the width of the sample. A slit was not used on the receiving side. The Cu-Kα radiation point source was operated at 40 kV and 200 mA. The scan was conducted from 3° to 32° (2θ) in steps of 0.02° and a counting time of 40 s, with β-axis rotation (20 rpm) during data collection. The data were analyzed using SmartLab studio II X64 version 4.2.111.0 (Rigaku Corporation).

### 2.7. Differential Scanning Calorimetry (DSC)

The heat flow profiles of AntiY_5_R, NaTC, their physical mixtures, and the co-amorphous systems were measured by differential scanning calorimetry (DSC) using a Discovery DSC (TA Instruments Japan, Tokyo, Japan). Nitrogen gas was used as the purge gas at 50 mL/min, and the instrument was calibrated with an indium standard. Because the profiles of AntiY_5_R and the physical mixtures of AntiY_5_R-NaTC (9:1) to AntiY_5_R-NaTC (6:4) showed higher crystallization potency, their amorphous samples were prepared using the melt quenching method.

Approximately 2–6 mg of AntiY_5_R, NaTC, and physical mixtures of AntiY_5_R-NaTC (5:5) to AntiY_5_R-NaTC (1:9) were weighed into a Tzero aluminium pan and sealed with a Tzero lid. The sample was then melted by heating to 275 °C at 20 °C/min, followed by cooling to −50 °C at −50 °C/min to investigate its melting point and crystallization tendency. The endpoint of heating was set before the rapid weight reduction observed in the TG/DTA measurements. A second heating step was also performed to investigate the heat flow properties of the amorphous form.

The melt-quench method was employed to determine the glass transition temperature of AntiY_5_R and the physical mixtures of AntiY_5_R-NaTC (9:1) and AntiY_5_R-NaTC (6:4). The samples were weighed into a pan, sealed using the same procedure described above, and melted on a hot plate at 275 °C for 10 s. The samples were immediately immersed in liquid nitrogen for 10 s. The cooled samples were then dried in desiccators containing silica gel for 1 min. The dried samples were then melted by heating to 275 °C at 20 °C/min to investigate the thermal behavior of the amorphous form.

The data were analyzed using Trios software (version 3.3.1; TA Instruments Japan). The onset of changes in the heat flow, exothermic, and endothermic peaks were designated as the glass transition temperature (*Tg*), crystallization temperature (*Tc*), and melting point (*Tm*), respectively.

### 2.8. Calculation of Theoretical Glass Transition Temperature

The theoretical values of *Tg* (K) for the mixtures of AntiY_5_R and NaTC were calculated using the Gordon-Taylor Equation [44,45] (1).

*Tg* = (*w*_1_·*Tg*_1_ + *K*·*w*_2_·*Tg*_2_)/(*w*_1_ + *K*·*w*_2_) (1)

where *w*_1_ and *w*_2_, and *Tg*_1_ and *Tg*_2_ are the weight fraction and glass transition temperature (*K*) of components 1 and 2, respectively. *K* is the curvature coefficient obtained from Equation (2).

*K* = *Tg*_1_·*ρ*_1_/*Tg*_2_·*ρ*_2_ (2)

where *ρ*_1_ and *ρ*_2_ mean densities of components 1 and 2, respectively.

### 2.9. Measurement of True Densities

The true densities of AntiY_5_R and NaTC were measured using Quantachrome Ultrapic 1200e (Anton Paar Japan K. K., Tokyo, Japan). The sample (0.3 mg) was weighed into a medium cell, and the volume (cm^3^) was measured under helium gas flow. The density (g/cm^3^) was calculated from the weight and volume. The densities of AntiY_5_R and NaTC were 1.346 ± 0.004 g/cm^3^ and 1.282 ± 0.002 g/cm^3^, respectively.

### 2.10. Fourier-Transform Infrared Spectroscopy

The molecular states of AntiY_5_R, NaTC, their physical mixtures, and their ball-milled mixtures were investigated by Fourier-transform infrared (FT-IR) spectroscopy using a VERTEX 70 spectrometer (Bruker Optics K.K., Tokyo, Japan). The numbers of scan time and resolution were 64 and 4 cm^−1^, respectively. The measurements focused on a wavenumber range in the carbonyl region, 1800–100 cm^−1^. The peak positions were assigned using ACD/Spectrus Processor 2019.2.2 software (Advanced Chemistry Development Inc., Toronto, ON, Canada).

### 2.11. In Vitro Dissolution Test

An in vitro dissolution test was performed using a μDISS Profiler (Pion Inc., Billerica, MA, USA) equipped with in situ fiber-optic UV probes and a mini-bath system for temperature and agitation control. The fiber optic probe assembly with a 20 mm pathlength tip was positioned at the center of each cylindrical vessel, and the tip was approximately 3 cm above the bottom. Powder samples of the physical mixture or co-amorphous AntiY_5_R-NaTC of 2 mg as AntiY_5_R were manually added to each vessel. The dissolution test was performed with 20 mL of pH 6.8 at 37 °C. The agitation speed was set at 300 rpm. Spectra were collected at predetermined time points, 5 s intervals for the first 10 min, followed by 20 s intervals for 50 min, and 1 min intervals for a further 120 min. The amount of dissolved AntiY_5_R was determined at 250 nm using an established calculation curve. The dissolution test was performed in triplicate for each formulation. The standard curves of 1.03 to 31.9 μg/mL showed a coefficient of variation ranging from 0.072 to 30% and squared correlation coefficients of over 0.999.

### 2.12. Isothermal Crystallization

The crystallization tendencies of co-amorphous AntiY_5_R-NaTC were investigated as follows. The samples were filled into a circular depression (diameter 3 mm, depth 0.1 mm) in an aluminium plate and were placed in two desiccators with silica gel. Each desiccator was stored at 25 °C or 40 °C for 1 month [42]. The samples in each desiccator were analyzed periodically using XRPD measurements.

## 3. Results and Discussion

### 3.1. Physicochemical Properties of Pure Components

**Figure 1:**
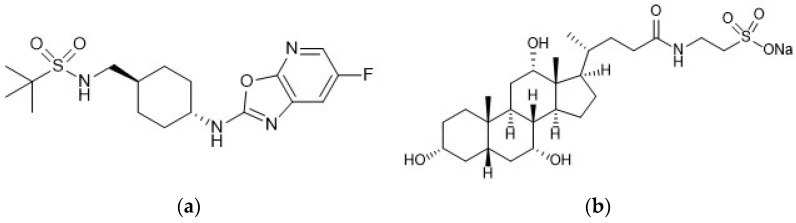
Chemical structures of (**a**) AntiY_5_R and (**b**) NaTC.

As shown in Figure 1a, AntiY_5_R has a sulfonamide group and the strongest acidic and basic pKa values calculated were 11.7 (sulfonamide group) and −0.1 (amine group), respectively, suggesting that the compound would practically be neutral. The solubility of AntiY_5_R in water, pH 1.2, and pH 6.8 buffer solutions was 2.6 ± 0.0, 9.0 ± 0.0, and 2.4 ± 0.0 μg/mL, respectively, indicating its low aqueous solubility. In the case of NaTC, the calculated strongest acidic and basic pKa values of taurocholic acid (free form of NaTC (Figure 1b) were 1.4 (sulfonic acid group) and −0.7 (amine group), respectively.

**Figure 2:**
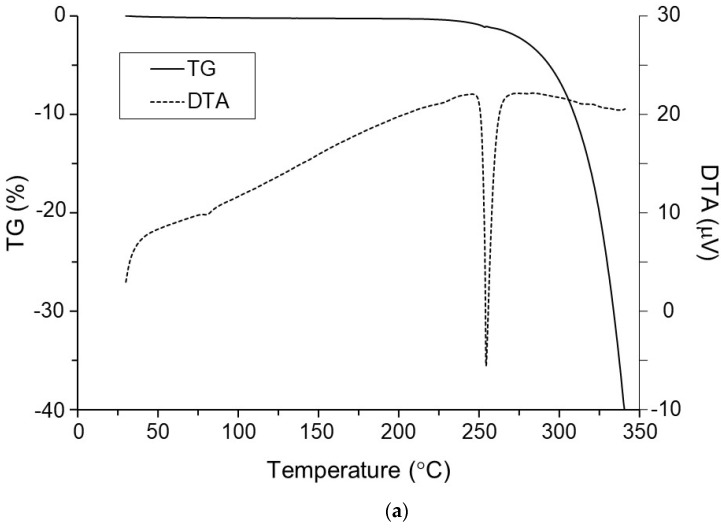

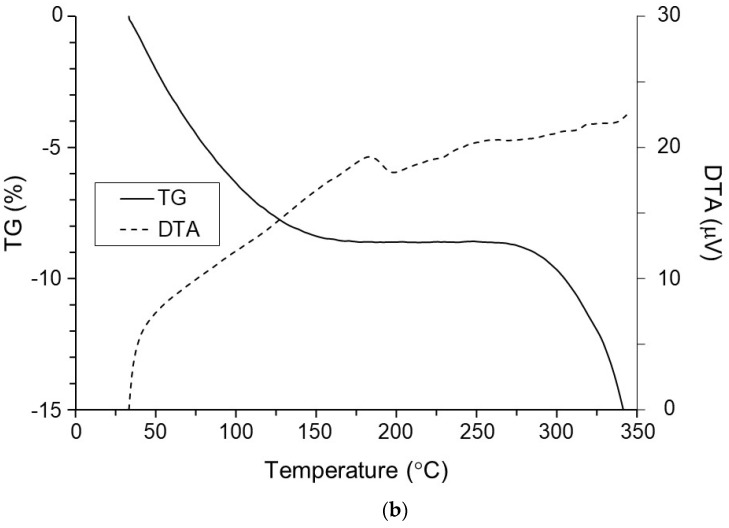
Typical TG/DTA profiles for (**a**) AntiY_5_R and (**b**) NaTC.

The TG/DTA profile of AntiY_5_R indicated that the *Tm* and thermal degradation of AntiY_5_R were 250.2 ± 0.2 °C and over 300.5 ± 1.1 °C, respectively (Table 1 and Figure 2a), suggesting that AntiY_5_R should be a brick dust compound [4,5]. On the other hand, Figure 2b indicates that the weight of NaTC was reduced by 7.1 ± 1.0% when heated to 100 °C, suggesting the desorption of water from the NaTC powder. Further heating did not change the weight of NaTC until approximately 270 °C, but a steep reduction in weight, reflecting thermal degradation, was observed at an onset temperature of 306.3 ± 4.1 °C (Table 1). The thermal stability of NaTC observed here is similar to or greater than that of amino acids [46,47]. The *Tm* of NaTC was unclear because a clear endothermic peak was not observed, as shown in Figure 2b.

**Figure 3:**
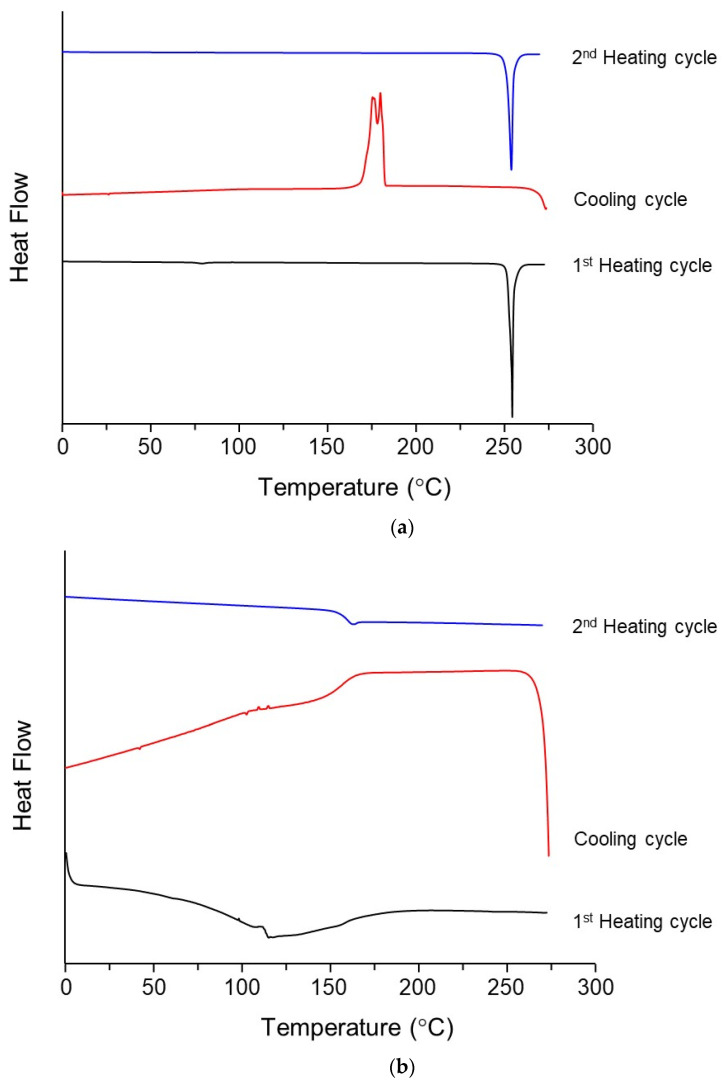
Typical DSC profiles of 1st heating, cooling and 2nd heating cycle for (**a**) AntiY_5_R and (**b**) NaTC.

DSC analysis was performed for the AntiY_5_R and NaTC (Figure 3 and Table 2). The *Tm* of 250.6 ± 1.2 °C was obtained for AntiY_5_R (Table 2), which was in agreement with that measured by TG/DTA. During the cooling cycle, an exothermic peak was observed at approximately 170 °C (Table 2), indicating that AntiY_5_R was crystallized. The crystallization tendency of the amorphous system was categorized into three classes based on the DSC profile as follows: Class I, High crystallization tendency; Recrystallization during the cooling cycle. Class II, Medium crystallization tendency; Recrystallization during 2nd heating cycle. Class III, Low crystallization tendency; No-recrystallization [48]. AntiY_5_R could be classified as “Class I”, since crystallization was observed during the cooling cycle, as shown in Figure 3a. In the 2nd heating cycle, the DSC profile was almost superimposed on that of 1st heating cycle; therefore, no information on *Tg* of AntiY_5_R was obtained in this study.

In the case of NaTC, as reported previously [41], the melting point and *Tg* of NaTC were not clear during the 1st heating cycle (Figure 3b), since a broad endothermic peak was only observed up to approximately 100 °C owing to water vaporization. In the 2nd heating cycle, the profile captured obvious glass transition behavior, resulting in *Tg* of 155.2 ± 0.6 °C (Table 2). In contrast, no endothermic peak was observed in the 2nd heating cycle. These results suggest that NaTC would be amorphous and be categorized into “Class III” with low crystallization potency [48]. The high *Tg* value of NaTC observed in the current study indicates that NaTC is a promising small-molecule coformer with higher thermal stability than other commonly used small-molecule coformers, including cholic acid (*Tg*, 120 °C [39]), amino acids (*Tg*, approximately 40–70 °C [36]), citric acid (*Tg*, 11 °C [37]), and lactic acid (*Tg*, −60 °C [37]).

### 3.2. Glass Transition Behaviors of AntiY_5_R and Its Co-Amorphous Systems

**Figure 4:**
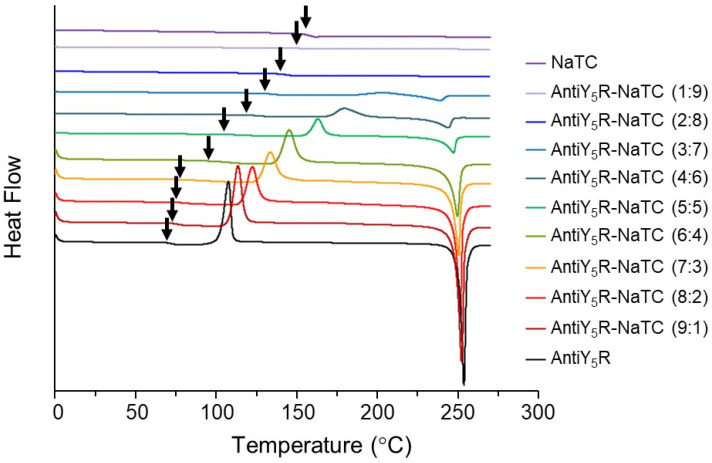
Typical DSC profiles of AntiY_5_R, NaTC and mixtures of AntiY_5_R and NaTC at different molar ratios. Amorphous samples of AntiY_5_R and AntiY_5_R-NaTC (9:1) to AntiY_5_R-NaTC (6:4) were prepared by the rapid cooling of their melted samples with liquid nitrogen. Amorphous samples of NaTC itself and AntiY_5_R-NaTC (5:5) to AntiY_5_R-NaTC (1:9) were prepared by cooling cycle during DSC measurement. The black arrows indicate the *Tgs*.

As the *Tg* of AntiY_5_R was not observed (Figure 3a), the melt-quench method was employed to determine the *Tg* of AntiY_5_R (Figure 4). The heating profile of AntiY_5_R, obtained by rapid cooling of melted AntiY_5_R with liquid nitrogen, successfully revealed that the value of *Tg* should be 69.5 ± 0.4 °C (Table 2).

To investigate the formation of co-amorphous AntiY_5_R with NaTC, the thermal behaviors of their mixtures were also measured by DSC analysis. Figure 4 also shows the DSC profiles of mixtures of AntiY_5_R and NaTC at different molar ratios. In a preliminary study, since AntiY_5_R itself and the physical mixtures of AntiY_5_R-NaTC (9:1) to AntiY_5_R-NaTC (6:4) showed crystallization during cooling to −50 °C at −50 °C/min after heated to 275 °C, their amorphous samples were prepared by rapid cooling of the melted samples with liquid nitrogen. On the other hand, amorphous samples of NaTC and physical mixtures of AntiY_5_R-NaTC (5:5) to AntiY_5_R-NaTC (1:9) were obtained without crystallization by cooling the melted samples to −50 °C at −50 °C/min.

Figure 4 shows that each of the samples prepared above had a single *Tg*, meaning that the AntiY_5_R-NaTC at each molar ratio should be co-amorphous. It was also found that the *Tg* of the co-amorphous system increased as the NaTC ratio increased.

Recrystallization behavior was observed in the DSC profiles of AntiY_5_R and the mixtures of AntiY_5_R-NaTC (9:1) to AntiY_5_R-NaTC (3:7) over the glass transition. The *Tc* of the co-amorphous system also shifted to a higher temperature with an increase in the molar ratio of NaTC to AntiY_5_R, and then the *Tc* disappeared for AntiY_5_R-NaTC (2:8, 1:9), suggesting that they remained amorphous. These results indicate that NaTC has an antiplasticizing effect on AntiY_5_R. A similar tendency was previously reported for some co-amorphous systems [38,49].

The endothermic peak was observed in the DSC profiles of AntiY_5_R and mixtures of AntiY_5_R-NaTC (9:1) to AntiY_5_R-NaTC (3:7) after recrystallization. The peak would be assigned to the *Tm* of AntiY_5_R in the mixtures because it almost agreed with the *Tm* of crystalline AntiY_5_R (250.6 ± 1.2 °C) (Table 2). However, the *Tm* of AntiY_5_R in the mixtures tended to decrease as the NaTC molar ratio increased. A decrease in *Tm* was also reported in PASD studies, indicating miscibility between materials owing to interactions [50]. Therefore, the decrease in *Tm* observed here suggests an intermolecular interaction between AntiY_5_R and NaTC in the mixture.

**Figure 5:**
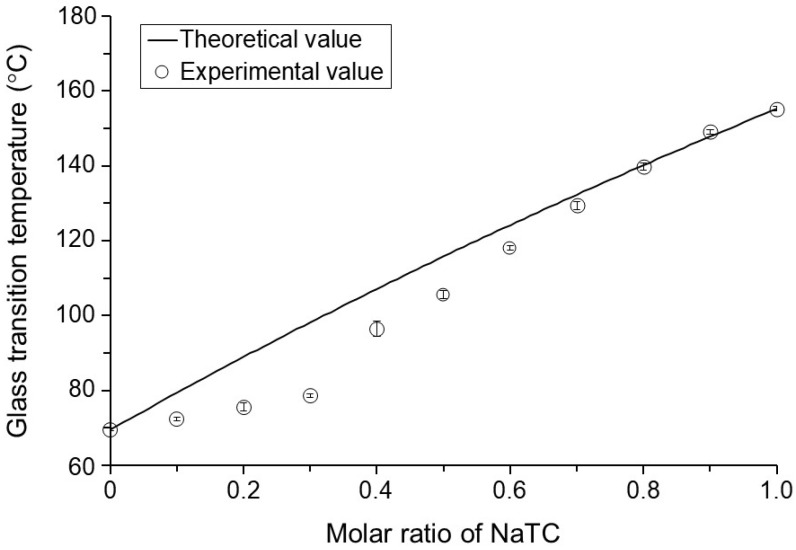
Comparison of *Tg* between the experimental and theoretical values for co-amorphous systems AntiY_5_R-NaTC at different molar ratios. Experimental values were expressed as the mean with standard deviation of three experiments. Keys: ○, the experimental values. Solid line indicates the theoretical values calculated by the Gordon-Taylor equation.

Since intermolecular interactions between AntiY_5_R and NaTC were suggested in their mixtures (Figure 4), *Tg* values obtained for the co-amorphous AntiY_5_R with NaTC were compared with theoretical values calculated using the Gordon–Taylor equation (Figure 5). The experimental data indicated that the increase in the NaTC ratio in the co-amorphous system increased the *Tg* from 69.5 ± 0.4 °C (AntiY_5_R) to 149.0 ± 0.5 °C (AntiY_5_R-NaTC (1:9)), which was higher than *Tg* values reported for co-amorphous systems comprising drugs with small-molecule coformers with *Tgs* similar to AntiY_5_R [34,35]. The theoretical value of *Tg* increased in proportion to the NaTC ratio. It clarified that the experimental data had a negative deviation from the theoretical values for the co-amorphous systems containing up to 70% NaTC at molar ratios. Since it has been reported that the deviation in *Tgs* values obtained experimentally from the theoretical values indicates the presence of intermolecular interactions between the components [35,49], it was considered that AntiY_5_R and NaTC interacted with each other even in the amorphous state. Furthermore, it has also been reported that the increase in *Tg* and intermolecular interactions with polymers could inhibit the crystallization of drugs and stabilize an amorphous state during storage in several PASD preparations [45,51,52]. Therefore, the co-amorphous system of AntiY_5_R with NaTC would be quite stable, and the amorphous state might be maintained even under accelerated conditions at 40 °C.

### 3.3. Preparation of the Co-Amorphous Systems

**Figure 6:**
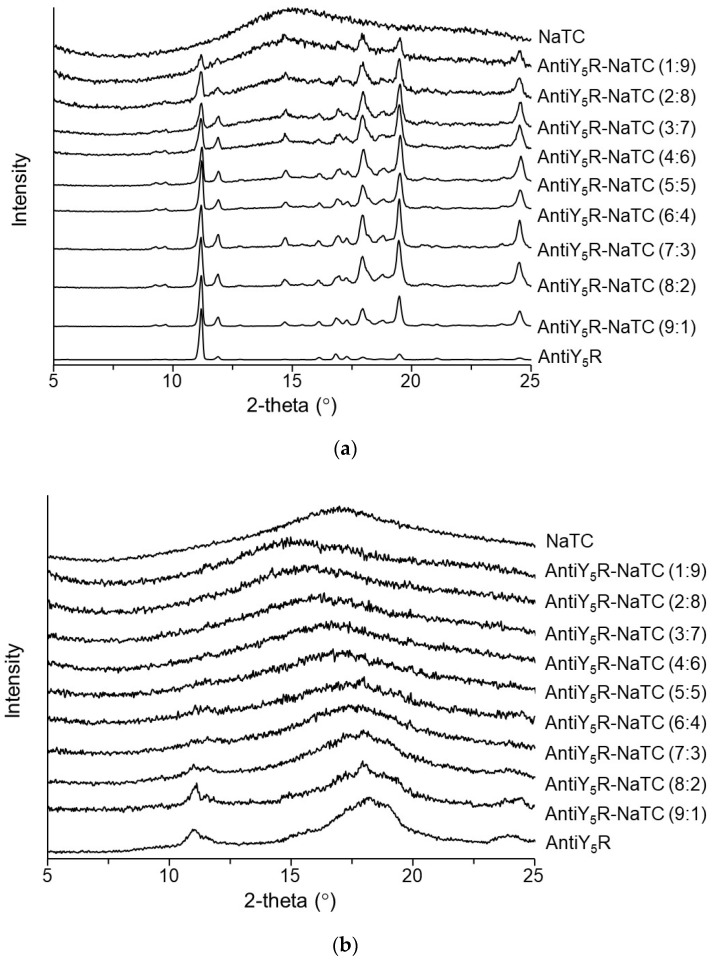
XRPD patterns of AntiY_5_R, NaTC and mixtures of AntiY_5_R and NaTC at different molar ratios. Typical patterns of XRPD for (**a**) before ball milling and (**b**) after 180 min ball milling.

We then attempted to prepare co-amorphous systems consisting of AntiY_5_R and NaTC from AntiY_5_R-NaTC (9:1) to AntiY_5_R-NaTC (1:9) using the ball milling method. AntiY_5_R, NaTC, and their physical mixtures in the corresponding ratios were ball-milled. Figure 6a,b shows the XRPD patterns before and after 180 min of ball-milling, respectively. NaTC showed a halo pattern even before milling, indicating that the raw material was amorphous, supporting the results obtained in Figure 2b, Figure 3b and Figure 4. In the case of AntiY_5_R, Figure 6a indicates that AntiY_5_R was in the crystal form for all the physical mixtures. On the other hand, Figure 6b shows that the mixtures of AntiY_5_R-NaTC (1:9) to AntiY_5_R-NaTC (5:5) exhibited typical halo patterns, suggesting the formation of co-amorphous systems. As for other preparations from AntiY_5_R-NaTC (6:4) to AntiY_5_R-NaTC (9:1), the XRPD patterns showed the possible remaining crystalline reflections in the diffractograms, although the XRPD pattern of AntiY_5_R provided a halo-like pattern, suggesting that the mixtures would still contain crystalline. Since ball-milled AntiY_5_R provided a halo-like pattern, suggesting partial amorphization, we preliminarily examined its aqueous solubility (4.6 ± 0.5 μg/mL) and found that it was slightly higher than, but similar to, that of the crystal. The results obtained here revealed that the ball-milling method could be useful for converting brick dust molecules to a co-amorphous system and that mixtures containing over 50% NaTC at a molar ratio should be employed as co-amorphous systems for further studies.

### 3.4. Physical Stability of the Co-Amorphous Systems

Because the amorphous state of the co-amorphous system should be stably maintained during storage for pharmaceutical dosage, the crystallization behavior of the co-amorphous AntiY_5_R-NaTC containing over 50% NaTC was investigated when stored at 25 °C or 40 °C. All samples were stable and showed no crystallization behavior during 1 month-storage at both temperatures. XRPD analysis revealed that each sample stored for 1 month provided a halo pattern, indicating that the co-amorphous system prepared with NaTC as a coformer has an excellent ability to avoid isothermal crystallization.

### 3.5. Fourier-Transformed Infrared Spectra of the Co-Amorphous Systems

**Figure 7:**
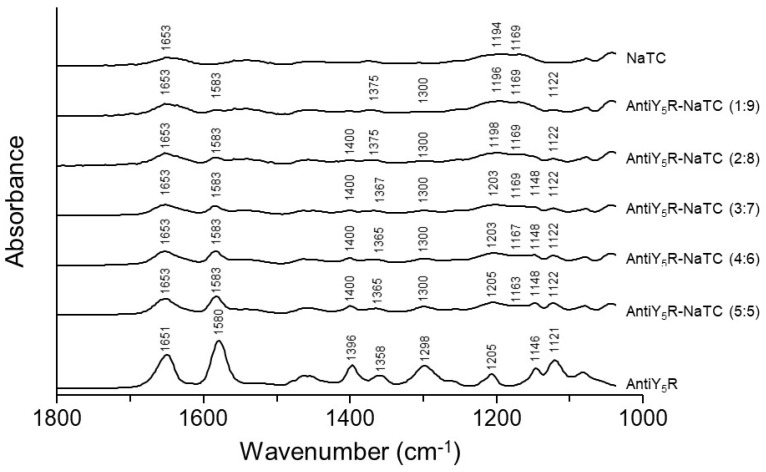
Typical FT-IR spectra of AntiY_5_R, NaTC and the co-amorphous systems comprised of AntiY_5_R and NaTC from AntiY_5_R-NaTC (1:9) to AntiY_5_R-NaTC (5:5).

Since the intermolecular interaction between AntiY_5_R and NaTC was suggested even in the amorphous state (Figure 5), possible interactions were investigated in detail using FT-IR analysis (Figure 7). The FT-IR spectra of NaTC showed broad peaks that were assigned to the C=O of the amide I band at 1653 cm^−1^ and the S=O of sulfonate stretching at 1194–1169 cm^−1^ [53]. AntiY_5_R showed the peaks of the C=N of oxazolopyridine symmetric stretching at 1651 cm^−1^, N-H_2_ in the plane band at 1580 cm^−1^, CH_3_ of tertiary butyl group symmetric deformations at 1396 cm^−1^, S=O of sulfonamide asymmetric stretching at 1358 cm^−1^, C-O-C of oxazolopyridine stretching at 1298 cm^−1^, C-F stretching at 1205 cm^−1^, and C-H of oxazolopyridine bending at 1146 and 1121 cm^−1^. The peak assignments were based on previous reports [54,55,56,57,58,59].

The spectra of the AntiY_5_R-NaTC preparations showed a similar spectral pattern to that of AntiY_5_R, but the peak derived from the S=O of sulfonamide for AntiY_5_R shifted from 1358 cm^−1^ to 1375 cm^−1^, while the peak derived from the S=O of sulfonate for NaTC shifted from 1169 cm^−1^ to 1163 cm^−1^ as the molar ratio of NaTC increased in the co-amorphous system. It was previously reported that Na^+^ could interact with other molecules in addition to the molecule with which Na^+^ forms the sodium salt [60]. Moreover, AntiY_5_R and NaTC are not able to form salts based on the calculated pKa values because their ∆pKa (∆pKa = pKa (base) − pKa (acid)) value is lower than 3 [61]. Therefore, these spectral changes suggest that the S=O of sulfonamide in AntiY_5_R and the S=O of sulfonate in NaTC might interact with each other through Na^+^.

### 3.6. Dissolution Studies of Co-Amorphous Systems

**Figure 8:**
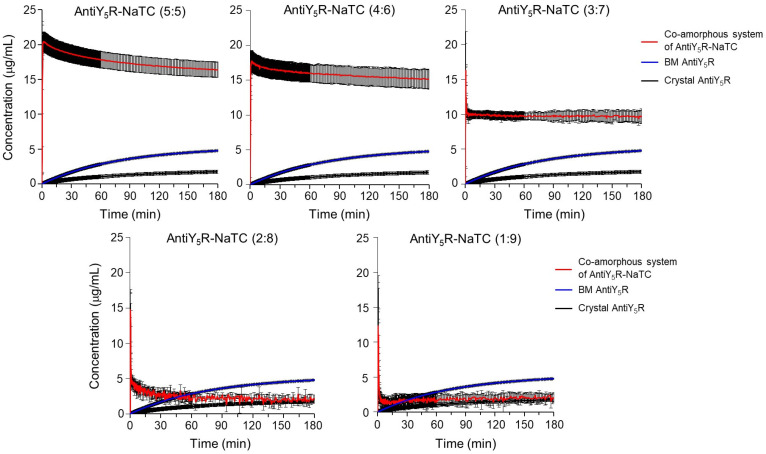
Powder dissolution profiles of crystalline AntiY_5_R, AntiY_5_R prepared by ball milling method (BM), co-amorphous systems from AntiY_5_R-NaTC (5:5) to AntiY_5_R-NaTC (1:9) prepared by BM. Results were expressed as the mean with standard deviation of three experiments. Keys: Red, blue and black solid line indicates the dissolution profile of the co-amorphous system, ball-milled AntiY_5_R and crystalline AntiY_5_R, respectively.

To investigate the potential supersaturation of AntiY_5_R promoted by co-amorphous AntiY_5_R-NaTC, a powder dissolution test was performed for pure crystalline AntiY_5_R, ball-milled AntiY_5_R, and co-amorphous AntiY_5_R-NaTC (5:5) to AntiY_5_R-NaTC (1:9) under non-sink conditions at pH 6.8 (Figure 8 and Table 3). Crystalline AntiY_5_R exhibited a concentration of 1.7 ± 0.1 μg/mL at the 180 min (Table 3), which was almost in accordance with the solubility (2.4 ± 0.0 μg/mL). The ball-milled AntiY_5_R dissolved more rapidly than the crystal, resulting in 4.8 ± 0.1 μg/mL (Table 3) higher than the solubility in pH 6.8 (2.4 μg/mL). The difference in the dissolution properties of the crystal and ball-milled AntiY_5_R could be responsible for the amorphous AntiY_5_R contained in the ball-milled sample, which was suggested in the XRPD pattern (Figure 6b). The dissolution profiles of the co-amorphous AntiY_5_R-NaTC preparations from 5:5 to 3:7 were much higher than those of crystalline or ball-milled AntiY_5_R. Specifically, the 5:5 preparation achieved the peak concentration, 20.5 ± 1.4 μg/mL (Table 3), which was 8.5-fold higher than the solubility in pH 6.8, 12.1-fold higher than the dissolved concentration for the crystal, and 4.3-fold higher than that for the ball-milled sample at the 180 min. Then the concentration gradually decreased and reached 16.4 ± 1.1 μg/mL at 180 min (Table 3), which was 6.8-fold, 9.6-fold and 3.4-fold higher than the solubility at pH 6.8, dissolved concentration for the crystal and that for ball-milled AntiY_5_R at 180 min, respectively. These results indicate that the coamorphization of AntiY_5_R with NaTC by ball milling is a promising way to enhance the solubility of brick dust molecules.

At the same time, however, an increase in the NaTC ratio in the system decreased the maximum dissolved concentration of AntiY_5_R. Specifically, the three preparations of 3:7, 2:8, and 1:9 AntiY_5_RNaTC showed an abrupt decrease in concentration immediately after the peak, and the 2:8 and 1:9 preparations almost fell down to the dissolved concentration of the crystal at 180 min. Vinarov et al. proposed that the addition of bile salts into the drug solution containing micelles would result in a decrease in drug dissolution because bile salts could disrupt the micelles by depriving surfactant molecules from the micelles by forming mixed micelles, and the solubilizing capacity would be lower than that of micelles [62]. Therefore, we thought that an excess of NaTC did not contribute to the generation of the co-amorphous form with AntiY_5_R, which may interfere with the coamorphization and/or dissolution of AntiY_5_R by depriving NaTC, contributing to the formation of the co-amorphous AntiY_5_R-NaTC. However, our preliminary study indicated that the addition of NaTC at 2.3 mM or 20 mM, more than the critical micelle concentration, gradually increased the dissolved concentration of AntiY_5_R from AntiY_5_R-NaTC (5:5) and achieved around 23 μg/mL, around 9.6-fold higher than the crystal solubility, at 180 min. These results suggest that a surplus of NaTC and/or micelles formed by NaTC does not necessarily suppress the dissolution of AntiY_5_R. Therefore, the reason why a larger amount of NaTC in the co-amorphous system decreased the extent of supersaturation of AntiY_5_R remains unclear and should be clarified in future studies.

## 4. Conclusions

In the current study, we attempted to prepare a stable co-amorphous system for a brick dust molecule, AntiY_5_R (a novel neuropeptide Y_5_ receptor antagonist), by utilizing NaTC, which has a relatively high *Tg*, as a coformer. We successfully prepared the co-amorphous system AntiY_5_R-NaTC at equal molar ratios, which maintained its amorphous state at 25 °C and 40 °C for 1 month and significantly improved the aqueous solubility of AntiY_5_R. A possible intermolecular interaction between AntiY_5_R and NaTC would contribute to the stability of the co-amorphous system. These results suggest that the utilization of a coformer with a high *Tg,* such as NaTC, is a promising strategy for preparing a stable co-amorphous system to improve the solubility of brick dust-like molecules. On the other hand, the reason why the increase in the NaTC ratio in the co-amorphous system decreased the dissolution property remains to be clarified. We would like to figure out the mechanisms underlying the symptom and to further improve the co-amorphous system by utilizing some polymers in a future study.

## Figures and Tables

**Table 1:** Thermal properties obtained by TG/DTA analysis for AntiY_5_R and NaTC.

Compounds	*Tm* (°C)	*Tdeg* (°C)
AntiY_5_R	250.2 ± 0.2	>300.5 ± 1.1
NaTC	N.D.	>306.3 ± 4.1

*Tm* and *Tdeg* mean the melting point and thermal degradation temperatures, respectively. N.D. mean “not detected”. Results are expressed as the mean ± S.D. of three experiments.

**Table 2:** Thermal properties obtained by DSC analysis for AntiY_5_R and NaTC.

Compounds	*Tm* (°C)	*Tg* (°C)	Crystallization (°C)
AntiY_5_R	250.6 ± 1.2	69.5 ± 0.4 ^1^	ca 170
NaTC	N.D.	155.2 ± 0.6 ^2^	N.D.

*Tm* and *Tg* mean the melting point and glass transition temperature, respectively. N.D. mean “not detected”. Results are expressed as the mean ± S.D. of three experiments. ^1^ melt-quench method. ^2^ 2nd heating cycle.

**Table 3:** Dissolution concentrations of AntiY_5_R from its crystalline, ball-milled AntiY_5_R and co-amorphous systems from AntiY_5_R-NaTC (5:5) to AntiY_5_R-NaTC (1:9).

Sampling Time	Crystalline (µg/mL)	Ball-Milled Crystalline (µg/mL)	AntiY_5_R-NaTC (Molar Ratio) (µg/mL)				
			5:5	4:6	3:7	2:8	1:9
Within 2 min ^1^	Not applicable	Not applicable	20.5 ± 1.4	17.7 ± 1.5	16.3 ± 0.8	14.8 ± 2.3	12.3 ± 6.2
180 min	1.7 ± 0.1	4.8 ± 0.1	16.4 ± 1.1	15.1 ± 1.4	9.8 ± 1.0	2.1 ± 0.5	2.1 ± 0.7

Results are expressed as the mean ± S.D. of three experiments. ^1^ The peak concentration of AntiY_5_R was observed within 2 min for the co-amorphous systems.
